# Instruments for Evaluating the Nutritional Status of Cancer Patients Undergoing Antineoplastic Treatment: A Scoping Review

**DOI:** 10.3390/nursrep14020099

**Published:** 2024-05-23

**Authors:** Erik Medina Cruz, Natacha Palenzuela Luis, Natalia Rodríguez Novo, Miriam González Suarez, Raquel Casas Hernández, María Mercedes Novo Muñoz

**Affiliations:** 1 University Hospital of the Canary Islands, 38320 Santa Cruz de Tenerife, Spain; natacha.palenzuela@universidadeuropea.es (N.P.L.); emedcruw@gobiernodecanarias.org (M.G.S.); 2 Departamento de Enfermería, Facultad de Ciencias de la Salud, Universidad de La Laguna, 38200 Santa Cruz de Tenerife, Spain; nrodrigu@ull.edu.es (N.R.N.); mernov@ull.edu.es (M.M.N.M.); 3 Nuestra Señora de La Candelaria University Hospital, 38010 Santa Cruz de Tenerife, Spain; rcasherw@gobiernodecanarias.org

**Keywords:** neoplasia, nutritional status, questionnaire, chemotherapy, immunotherapy

## Abstract

The use of validated tools to evaluate the nutritional status of the cancer patient provides guaranteed precision and reliability in their nutritional evaluation, ensuring that the information is accurate and reflects the patient’s situation. The aim of this study was to identify the valid and reliable instruments in the evaluation of the nutritional status of cancer patients with a diagnosis of solid tumor undergoing antineoplastic treatment (chemotherapy and/or immunotherapy). A scoping review was conducted to search for original articles published in scientific journals in English, Spanish, or Portuguese in the past five years. In order to identify potentially relevant documents, searches were performed in the following databases: SCOPUS, WOS, CINAHL, MEDLINE, BVS, and PUBMED. DECS-MeSH descriptors and Boolean operators were used. In addition, the Arksey and O’Malley protocol, the Joanne Briggs Institute (JBI) method, and the flow chart of the Preferred Information Elements for Systematic Reviews and Meta-Analyses, known as PRISMA, were followed. The initial search strategy identified a total of 164 references, which were examined successively, leaving a final selection of ten studies. It was found that the most used instrument for nutritional evaluation was the Patient-Generated Subjective Global Assessment (PG-SGA). Other questionnaires also stood out such as the Mini Nutritional Assessment (MNA), the Malnutrition Universal Screening Tool (MUST), the Nutritional Risk Screening (NRS 2002), and the Functional Assessment of Anorexia/Cachexia Therapy (FAACT). The variation in the tools used ranges from subjective assessments to objective measurements, thus underlining the need for a comprehensive and individualized approach.

## 1. Introduction

The optimal treatment for cancer patients should be characterized by being comprehensive and multidisciplinary [1]. Within this context, nutritional assessment is vital, because malnutrition in cancer patients not only has consequences at a physical level, but also psychologically, in addition to being associated with a worse prognosis of the disease and a decrease in health-related quality of life (HRQoL) [2,3]. HRQoL is the subjective evaluation of how health status, health care, and health promotion influence an individual’s ability to function and their well-being. The most significant dimensions in HRQoL are “social, physical and cognitive functioning, mobility, personal care and emotional well-being”, according to Schumakel and Naughton [4].

Although there are different definitions of cancer, all of them, including that of the World Health Organization (WHO), have common characteristics in referring to cancer as a group of conditions arising from the uncontrolled proliferation of abnormal cells in various tissues or organs of the body. This proliferation can exceed the normal limits of cell growth and result in the invasion of neighboring tissues, as well as spread to distant organs, a process known as metastasis [5].

Cancer is one of the main causes of mortality worldwide and it is estimated that 9.7 million deaths were related to this pathology in 2022. It is expected that more than 16 million people will die from cancer in 2040. Among the different types of cancer, lung cancer has the highest mortality, followed by colorectal, liver, stomach, and breast cancer [6]. Furthermore, in line with the international panorama, in the Canary Islands during 2022, 559 new cases were diagnosed per 100,000 inhabitants, and breast cancer, followed by prostate and colorectal cancer, was the most frequently diagnosed type [7].

Taking into account the above data, it is important to highlight that, statistically speaking, patients with pancreatic, gastrointestinal, or head and neck cancer have a higher risk of malnutrition, compared to patients with breast cancer [8,9,10], all of which are classified as solid tumors, in other words, cancers with a mass of solid tissue [11]. Based on this premise, the prevalence of malnutrition ranges between 25 and 70% in patients with an oncological diagnosis, depending on the population studied, varying not only in the location of the tumor but also the stage of the disease, adding to the side effects of antineoplastic treatment [8,9]. These treatments include chemotherapy (antineoplastic treatment that acts at a systemic level, that is, not only on the neoplastic cells, but also on the cells of the body that have the capacity to divide) [12] and immunotherapy (treatment responsible for stimulating the patient’s immune system to recognize cancer cells and stop their growth or destroy them, unlike conventional therapies that act directly on the tumor) [13].

Therefore, malnutrition associated with neoplastic diseases is understood as an imbalance between nutrient intake and needs. It is a very common problem among cancer patients that results in a deterioration in quality of life (QoL) and a worse prognosis for the disease [14,15]. In addition, the loss of body weight can lead to greater toxicity from chemotherapy, as well as lead to and prolong hospital stays, thereby increasing economic expenditure in relation to treatment [8,10,14].

Despite the high prevalence of oncological malnutrition and knowing that it causes increased mortality, it is usually underdiagnosed [10,16].

Inadequate nutrition is clearly associated with a worse prognosis and survival and, consequently, worse QoL [9,10,16]. This is why different institutions such as the American Society for Parenteral and Enteral Nutrition (ASPEN) and the European Society for Clinical Nutrition and Metabolism insist on the importance of nutrition detection and intervention in oncology patient populations [8,9,10,11,12,13,14,15]. Thus, several studies have demonstrated the benefits of a correct approach to cancer patients in reference to early nutritional support since it improves the QoL of patients diagnosed with cancer [1,8,15,16].

Therefore, the use of valid and reliable tools in the assessment of nutritional status makes it possible to guarantee accuracy and reliability in the holistic nutritional evaluation of the patient. In addition, it facilitates communication between health professionals since it provides a common language to describe nutritional status, making multidisciplinary work in patient care easier [17,18].

The present study aims to identify the valid and reliable instruments in the evaluation of the nutritional status of cancer patients with a diagnosis of solid tumor undergoing antineoplastic treatment (chemotherapy and/or immunotherapy).

## 2. Materials and Methods

A scoping review was conducted, using DECS-MeSH descriptors and Boolean operators. In addition, the Arksey and O’Malley protocol [19], the Joanne Briggs Institute (JBI) method [20], and the flow chart of the Preferred Information Elements for Systematic Reviews and Meta-Analyses, known as PRISMA, were applied [21]. This study was prospectively registered in the Open Science Framework (OSF) with registration number 10.17605/OSF.IO/ABRQK.

### 2.1. Selection Criteria in the Study

Original articles published in scientific databases in English, Spanish, or Portuguese over the past five years were included. Studies evaluating the nutritional status of patients aged 18 years or older, undergoing active treatment with chemotherapy and/or immunotherapy due to a diagnosis of solid tumor were included.

Summaries, editorials, comments, and book reviews were excluded, as were studies conducted with children and/or adolescents.

Search strategy:

The review was carried out in five phases, taking into account the Arksey and O’Malley criteria [19]:

- Phase 1: Identification of the research question.
- Phase 2: Identification of relevant studies.
- Phase 3: Selection of studies taking into account the inclusion and exclusion criteria.
- Phase 4: Registration and presentation of data.
- Phase 5: Compilation, summary, and communication of the results.

A first search was performed with the intention of contextualizing the topic of interest and locating previous publications that answered the research question; databases such as PubMed and ScienceDirect were accessed for this purpose. Furthermore, this made it possible to identify possible descriptors that would be subsequently used in the search for the desired evidence (“neoplasia”, “cancer”, “nutritional assessment”, “questionnaire”, and “chemotherapy”).

A preliminary search was carried out in different databases (DB): SCOPUS, WOS, CINAHL, MEDLINE, BVS, and PUBMED. The search strategies were reviewed by two collaborators and subsequently refined through team discussions. Table 1 shows the search strategy for 19 December 2023, used in the different databases, as well as the Boolean operators and DECS-MeSH descriptors used.

### 2.2. Data Analysis

The search began with a selection of works according to title, abstract, and keywords, with the aim of choosing those works that met the established selection criteria. Studies included in this phase and those without sufficient information to determine their selection underwent a second phase of full text review.

A template developed by JBI was created for the data extraction process to detail the characteristics and results of the included studies [20]. The following information was selected from each article: authors, year of publication, country or city, objective, population, methodology, and main findings.

## 3. Results

**Figure 1:**
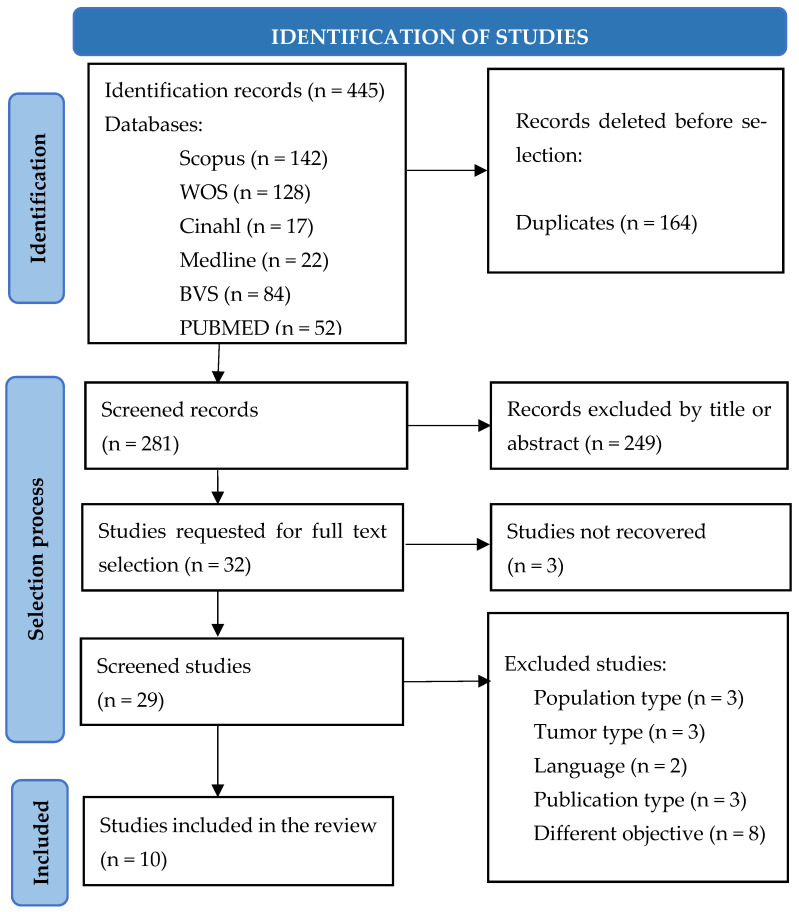
Article selection following the PRISMA 2020 method flow chart.

In the initial search, a total of 445 articles were identified in the different databases, and duplicates were subsequently eliminated and selected. Taking into account that the objective of this review was to find out the valid and reliable instruments in the evaluation of the nutritional status of cancer patients with a diagnosis of solid tumor undergoing antineoplastic treatment (chemotherapy and/or immunotherapy), in the end, ten articles were included, following the PRISMA flow chart, which is shown in Figure 1.

It was observed, after the analysis of the ten articles, that the types of studies were mainly descriptive [22,23,24,25,26,27,28], in addition to a cohort study [29], another prospective and analytical study [30], and finally, a systematic review [31]. Table 2 presents a summary of the most relevant aspects of each of the articles (authors, year of publication, country or city, objective, population, methodology, and main findings).

## 4. Discussion

### 4.1. Instruments for Nutritional Evaluation

In the majority of the studies reviewed, the Patient-Generated Subjective Global Assessment (PG-SGA) was used to assess the nutritional status of cancer patients, in addition to using this tool to correlate it with other variables of the cancer patients, such as QoL, as Badrasaw et al. [23] or Kim et al. [30] did in their analytical studies, to observe the effect on the nutritional status of patients with pancreatic and bile duct cancer undergoing chemotherapy and nutritional intervention by giving nutritional supplements.

Beukers et al. [31], in their systematic review, associated the results of PG-SGA with tolerance to the systemic treatment that was being administered to the patients. On the other hand, Hasegawa et al. [29] used another version of this questionnaire, the Subjective Global Assessment (SGA), where the part completed by the patient is omitted. Although the authors did not mention it explicitly, it is possible to infer that this choice was due to the specific focus of their study. Their main interest lay in weight loss and physical assessment, prioritizing these aspects over the symptoms manifested by the patients. This aligns with the objective of the cohort, which focused on protein intake as a prognostic factor during chemotherapy.

Despite the above, studies such as that of Bauer et al. [32] compared the sensitivity and specificity between the PG-SGA and the simple version of SGA. As a result, they found that the former method had a greater sensitivity and specificity, 98% and 82%, respectively. Furthermore, the Spanish Nutrition and Cancer working group of the Spanish Society of Basic and Applied Nutrition also selected this methodology as the most appropriate one for nutritional assessment in cancer patients [33].

Hettiarachchi et al. [26] evaluated the agreement between the PG-SGA and another nutritional screening questionnaire, the Malnutrition Universal Screening Tool (MUST). Their results showed how both questionnaires have a high level of agreement in detecting the risk of malnutrition in patients receiving chemotherapy. The authors emphasized the importance of selecting appropriate and reliable tools to evaluate nutritional status and said that the amount of time spent by the health professional is a limitation for the use of PG-SGA, compared to the speed and simplicity of the MUST questionnaire.

The Spanish Society of Radiotherapy Oncology (SEOR), in contrast to the authors of the above mentioned studies, has pointed out that, for cancer patients, the MUST tool has proven not to be useful due to its low sensitivity and specificity. This is why SEOR and the Spanish Society of Clinical Nutrition and Metabolism (SENPE) have published a consensus guide for the management of nutrition in cancer patients, in which the Malnutrition Screening Tool (MST) questionnaire is recommended as a screening method for cancer patients. Furthermore, both the MST and PG-SGA effectively predict the nutritional status of the patient, and, in addition, the MST questionnaire has been validated both for inpatients and outpatients [34,35].

On the other hand, Hamdan et al. [25] did not use the PG-SGA to measure the risk of malnutrition, but, instead, they used the Nutritional Risk Screening (NRS 2002), in addition to the Functional Assessment of Anorexia/Cachexia Treatment (FAACT) questionnaire. Their results showed that the differences between the scores of the two questionnaires were not significant in terms of their correlation. Regarding the use of the NRS 2002 questionnaire, and taking into account that one tool or another will be applied depending on the scientific society or the healthcare process that is taken as a reference, the European Society of Clinical Nutrition and Metabolism (ESPEN) recommends using this screening method for inpatients and MUST for outpatients [35].

In a German study conducted by Sonneborn-Papakostopoulos et al. [28], they used the Mini Nutritional Assessment (MNA) questionnaire as a tool to evaluate the nutritional status of cancer patients. Like those previously mentioned, it has measurable elements to evaluate weight loss during defined periods of time and the presence of symptoms associated with the disease and/or treatment. It should be noted that although this type of questionnaire does not require biochemical determinations or anthropometric parameters, ESPEN recommends it only for elderly patients, since it has been validated for people over 65 years of age in several countries, including Spain [36].

### 4.2. Body Mass Index (BMI) and Biochemical Parameters in the Evaluation of the Nutritional Status of Cancer Patients

In most studies, together with one or several validated nutritional screening tools, an objective assessment is frequently performed using BMI and biochemical parameters that are used in routine clinical practice, and they can be considered as good indicators of nutritional status, as is the case of albumin or C-reactive protein (CRP).

In relation to BMI, it should be noted that it has not been shown to be a good indicator of malnutrition in any of the studies selected for this review. In their results, Hettiarachchi et al. [26] showed that BMI wrongly classified 37.6% of patients as having good nutritional status, demonstrating the low sensitivity of this parameter and a weak association with PG-SGA. The same occurs in Milani et al. [27], who highlighted in their study carried out in Brazil that BMI should not be considered as the only indicator of nutritional evaluation, due to the discrepancies found between the nutritional diagnosis generated by the validated instrument they used, PG-SGA, with 37.4% being considered as moderately malnourished and 31.3% as severely malnourished, compared to 9.1% according to BMI. On the other hand, Ferigollo et al. [24] pointed out that 77% of patients in the PG-SGA had low weight and 40% had severe weight loss; however, the evaluation of nutritional status using BMI highlighted the prevalence of normal weight and overweight in the patients. Similarly, Hamdan et al. [25] reported that there was no relevant association between the results of BMI and the FAACT and the NRS test. In conclusion, BMI should not be used on its own for the diagnosis of malnutrition, since it has several limitations, among which the artificial increase in body weight due to the accumulation of fluid in the patient and its failure to distinguish between lean and fatty tissue. Added to all this is that the risk of malnutrition is sometimes masked by normal or higher BMI values, positioning it as an indicator with little potential to detect nutritional risk [18,37,38].

Among the biochemical parameters used by the studies in this review, those that stand out are CRP, albumin, lymphocytes, neutrophils, transferrin, hemoglobin, and cholesterol. There is a relationship in all of them between the alteration of analytical data and worse nutritional status. Hamdan et al. [25] highlighted that the majority of patients had low hemoglobin levels (76.3%), with no apparent relationship with nutritional status, unlike albumin, indicating that participants with lower levels than the established ones had a worse score in the FACCT and NRS 2002 questionnaire. The same applied in the study of Hasegawa et al. [29], when transferrin and total protein levels were added to this correlation. Similarly, but, on this occasion, taking the PG-SGA as an evaluation tool, Ferigollo et al. [24] reported an association between albumin and malnutrition, but not with lymphocyte count, despite the authors indicating, as part of their hypothesis, that reduced levels of albumin and lymphocytes are indicators of worse clinical outcomes. Finally, the study by Kim et al. [30], on nutritional intervention in pancreatic and bile duct cancer using biochemical parameters, did not show significant differences after eight weeks, unlike the association in weight gain, PG-SGA, and the perception of quality of life. In other words, the biochemical parameters in a patient with cancer have the disadvantage of being modified by factors specific to the disease, such as the inflammatory state. To this we should add the effects of antineoplastic treatment and the rest of the medications, since these can affect, for example, the lymphocyte count [2,17,18,38].

### 4.3. Antineoplastic Treatment and Nutritional Status

The studies selected for this review used chemotherapy treatments as the main antineoplastic agent. No studies with greater representativeness were identified in the approach to cancer patients with immunotherapy.

Hamdan et al. [25] and Hasegawa et al. [29] focused on the nutrition and functional status of cancer patients undergoing chemotherapy. These studies highlighted the impact of nutritional support on the overall well-being and even the survival of patients. They suggested that optimizing nutritional intake, particularly protein, during chemotherapy may be a predictor of better outcomes.

In their systematic review, Beukers et al. [31] examined the associations between outcome variables of nutritional screening methods and tolerance to systemic treatment in patients with colorectal cancer. Their results underline the importance of nutritional screening to predict tolerance to treatment, since significant relationships were found between malnourished patients with a greater probability of toxicity in antineoplastic treatments. This correlation was detected in both the PG-SGA and the MNA questionnaire, in addition to a significant association between weight loss and general toxicity.

### 4.4. Quality of Life and Nutritional Status

Nutritional status and its relationship with QoL are closely related; five studies included in this review referred to this, in addition to other publications in the scientific literature, which conclude that an adequate nutritional status positively influences QoL and, consequently, improves tolerance to treatments [39,40].

Except for Hamdan et al. [25], who used the Functional Assessment of Cancer Therapy Scale, General (FACT-G) questionnaire to measure quality of life, the rest of the studies used the European Organization for Research and Treatment of Cancer QoL Questionnaire C30 (EORTC-C30) [22,23,28,30]. Both questionnaires are classified as specific instruments in oncological pathologies [41].

In relation to the first tool mentioned, Hamdan et al. [25] observed that patients with malnutrition had lower final scores on the FACT-G questionnaire, without clarifying which of the scales was most affected (physical state, family and social environment, emotional state, or capacity for personal functioning).

Badrasawi et al. [23], on the other hand, used the EORTC-C30 questionnaire. The authors highlighted that malnourished patients had a lower functional status and a higher level of fatigue compared to well-nourished and slightly malnourished patients, taking into account that patients with more advanced cancer stages had a higher prevalence of malnutrition. Similarly, physical, emotional, and cognitive functions were significantly lower in patients with advanced stages of cancer. These findings confirm that the higher the stage of cancer, the worse the quality of life.

On the other hand, in their study on breast cancer, Adam et al. [22] also applied the EORTC-C30 tool to evaluate QoL. In it, they obtained the highest mean average score in cognitive functioning, while the most affected domain among the symptomatic scales was again fatigue in terms of malnutrition, as in the results published by Hasegawa et al. [29], who also reported that nausea and vomiting presented the highest scores. Regarding the global QoL scale, both studies said that the mean QoL level was lower in moderately and severely malnourished people compared to well-nourished people. Malnutrition decreased the scores of the functional scales, as well as the mean average level of QoL [22,29]. Adam et al. [22] concluded by highlighting the importance of nutritional evaluation, as malnutrition decreases the quality of life, fundamentally through its influence on muscle strength and the sensation of weakness and asthenia, due to the loss of muscle mass that it causes and its influence on the patient’s state of mind, inducing or intensifying depressive symptoms. This clinical situation increases the incidence of complications and the patient’s hospital stay, decreasing the time free of symptoms and independent life outside the hospital, and thus strongly contributing to the deterioration of their QoL [42,43,44].

The present review has several limitations. Firstly, no recent available research was found in Spain. This limitation is an indication of the need to promote the development of studies with similar characteristics since, from a scientific point of view, it would be interesting to investigate the nutritional problems and QoL of cancer patients. On the other hand, most of the included works are cross-sectional observational studies that do not allow inferences of causality, and the samples lack representativeness. Furthermore, all authors talk about chemotherapy as the only treatment; no results were shown with patients treated with immunotherapy. However, this systematic review would provide representative data if studies were carried out with a similar methodology. Finally, future research would expand on relevant concepts such as sarcopenia and cachexia in oncology patients due to their relevance to their nutritional status.

## 5. Conclusions

In conclusion, the studies discussed here collectively emphasize the multifaceted nature of nutritional assessment in cancer patients. The variation in tools used, ranging from subjective assessments to objective measurements, underscore the need for a comprehensive and individualized approach. The integration of various assessment methods and patient self-perception in terms of quality may improve the precision of nutritional interventions, ultimately benefiting the well-being and outcomes of patients diagnosed with cancer.

## Figures and Tables

**Table 1:** Search strategy in the different databases.

Database	Search Strategy	Search Date
SCOPUS	((cancer OR neoplasms) AND nutritional AND status AND (questionnaires OR surveys) AND (immunotherapy OR chemotherapy))	19 December 2023
WOS	(cancer OR neoplasm) AND nutritional status AND (questionnaires OR surveys) AND (immunotherapy OR chemotherapy) NOT surgery NOT children NOT hematology	19 December 2023
CINAHL	(cancer OR neoplasms) AND nutritional status AND (questionnaires OR surveys) AND (immunotherapy OR chemotherapy)	19 December 2023
MEDLINE	(cancer OR neoplasms) AND nutritional status AND (questionnaires OR surveys) AND (immunotherapy OR chemotherapy)	19 December 2023
BVS	(cancer OR neoplasms) AND nutritional status AND (questionnaires OR surveys) AND (immunotherapy OR chemotherapy)	19 December 2023
PUBMED	(cancer OR neoplasms) AND nutritional status AND (questionnaires OR surveys) AND (immunotherapy OR chemotherapy) NOT children NOT (hematology AND surgery)	19 December 2023

**Table 2:** Analysis of the ten articles selected for the scoping review.

Authors and Year	Country/City	Study Objective	Type of Study/ Methodology	Participants	Main Findings
Adam R et al. (2023) [22]	Ethiopia	To determine the relationship between nutritional status and quality of life among breast cancer patients receiving treatment.	Descriptive, observational, cross-sectional study	Patients with breast cancer undergoing treatment (n = 401)	Nutritional status was evaluated with the Subjective Global Assessment (SGA) questionnaire.
Badrasawi et al. (2021) [23]	Palestine	To determine the relationship between nutritional status and quality of life of cancer patients receiving chemotherapy.	Descriptive and cross-sectional study	Cancer patients receiving chemotherapy (n = 100)	The Subjective Global Assessment (SGA) questionnaire was used for the nutritional evaluation.
Ferigollo A et al. (2018) [24]	Brazil	To identify the nutritional status and factors associated with possible nutritional changes in cancer patients undergoing antineoplastic treatment.	Descriptive and cross-sectional study	Cancer patients undergoing antineoplastic treatment (n = 60).	The Patient-Generated Subjective Global Assessment (PG-SGA) questionnaire was used.
Hamdan M et al. (2022) [25]	Palestine	To determine the prevalence of malnutrition among cancer patients and evaluate the nutritional and functional status of cancer patients undergoing chemotherapy.	Descriptive and cross-sectional study	Cancer patients receiving chemotherapy (n = 132)	The Nutritional Risk Screening (NRS 2002) was used to evaluate the risk of malnutrition. The Functional Assessment of Anorexia/Cachexia Therapy (FAACT) questionnaire was used to determine functional status.
Hasegawa Y et al. (2021) [29]	Tokyo	To investigate nutritional status and longitudinal food intake during chemotherapy, and its relationship with survival, in newly diagnosed patients with unresectable pancreatic cancer.	Prospective cohort study	Patients with unresectable pancreatic cancer undergoing chemotherapy (n = 38)	Subjective Global Assessment (SGA) was used to evaluate the nutritional status of the patients.
Hettiarachchi J et al. (2018) [26]	Sri Lanka	To evaluate agreement between the Malnutrition Universal Screening Tool (MUST) and the Patient-Generated Subjective Global Assessment (PG-SGA) to detect the risk of malnutrition in medical oncology outpatients.	Observational and cross-sectional study	Cancer patients receiving chemotherapy (n = 100)	Two questionnaires were used to evaluate nutritional status: Malnutrition Universal Screening Tool (MUST) and Patient-Generated Subjective Global Assessment (PG-SGA).
Milani J et al. (2018) [27]	Brazil	To compare results of anthropometry and subjective nutritional evaluation applied to cancer patients.	Descriptive and cross-sectional study	Patients with cancer who are being treated with chemotherapy (n = 99)	The Patient-Generated Subjective Global Assessment (PG-SGA) was used.
Sonneborn-Papakostopoulos M et al. (2021) [28]	Germany	To evaluate oncology outpatients and the risk of malnutrition.	Descriptive and cross-sectional study	Cancer patients receiving chemotherapy (n = 109)	Nutritional status was evaluated with the Mini Nutritional Assessment (MNA) questionnaire.
Kim S et al. (2019) [30]	Korea	To investigate the beneficial effects of oral nutritional supplements (ONSs) in patients with pancreatic and bile duct cancer undergoing chemotherapy.	Analytical and prospective study	Patients with pancreatic and bile duct cancer undergoing chemotherapy treatment (n = 34)	Nutritional status was evaluated with the Patient-Generated Subjective Global Assessment (PG-SGA).
Beukers K et al. (2022) [31]	Netherlands	To systematically investigate which outcome variables of nutritional screening methods are associated with treatment tolerance in patients with colorectal cancer.	Systematic review	Patients with colorectal cancer undergoing treatment (n = 16)	Mini Nutritional Assessment, Nutritional Risk Index, and Patient-Generated Subjective Global Assessment (PG-SGA).

## Data Availability

Not applicable.

## Public Involvement Statement

There was no public participation in any aspect of this research.

## Guidelines and Standards Statement

This manuscript was written according to the Joanna Briggs Institute methodology for scoping review research.
